# Comparison of the Detection Rates of Different Diagnostic Methods for Primary Peripheral Lung Cancer

**DOI:** 10.3389/fonc.2021.696239

**Published:** 2022-03-17

**Authors:** Lijuan Sun, Chao Qin, Qun Fu, Shuangmin Hu, Wenfei Zhao, Hongyun Li

**Affiliations:** Department of Respiratory Medicine, The Fifth Affiliated Hospital of Zhengzhou University, Zhengzhou, China

**Keywords:** fiberoptic bronchoscope, peripheral lung cancer, CT-guided, methods of sampling, clinical research

## Abstract

**Objective:**

The present study aims to compare the detection rates of different diagnostic methods for primary peripheral lung cancer (PPLC).

**Methods:**

The detection rate and patient information were collected from a total of 359 cases of PPLC or a suspected diagnosis of lung cancer; among these, 186 cases were simultaneously treated with fibreoptic bronchoscopy, brush inspection and flush inspection, and 173 cases underwent a computed tomography (CT)-guided percutaneous lung biopsy (PNB). The positive detection rates of the different methods were compared.

**Results:**

In the detection of peripheral lesions (diameter of <5 cm), the CT-PNB had the significantly highest detection rate, followed by the combined basic method (fibreoptic bronchoscopy + brushing + flushing). The independent use of the three basic sampling methods showed a significantly lower detection rate compared with the combined use.

**Conclusion:**

In the diagnosis of peripheral lung cancer, the CT-PNB had the best detection rate; hence, it could be used in clinical practice for the diagnosis of such lesions.

## Introduction

Lung cancer is a common respiratory disease that presents with malignant tumours and has high morbidity and mortality rates (1). Primary peripheral lung cancer (PPLC) lesions occur below the tertiary bronchi and above the respiratory bronchioles (2); they are mostly adenocarcinomas (adenocarcinoma of the lung is more common than squamous cell carcinoma) (2, 3). In contrast to adenocarcinoma, squamous cell carcinoma is related to long-term smoking, ionizing radiation, environmental factors and occupational risk factors (4).

With the improvement of people’s health awareness in recent years, the number of people undergoing computed tomography (CT) screening has increased, as has the detection rate of early lung cancer (5, 6). There are many navigational techniques for reaching peripheral lung lesions, such as radial endobronchial ultrasound (R-EBUS) combined with a guide sheath pipe (GS) for transbronchial lung biopsy (TBLB), virtual bronchoscopic navigation, lung biopsy under X-ray line monitoring, lung point navigation, electromagnetic navigation bronchoscopy (ENB), multimodal augmented-reality lung navigation technology (e.g. Lungpro) and joint X-ray C-arm positioning advanced bronchoscopic transparenchymal nodule access technology for detecting lesional tissue (7).

These techniques compensate for the low positive rate of fibreoptic bronchoscopy, which is caused by the method’s inability to reach peripheral lung lesions; however, these techniques are not widely used. In our hospital, fibreoptic bronchoscopy combined with a variety of basic sampling methods, along with CT-guided percutaneous lung puncture biopsy (CT-PNB), lymph node biopsy and surgical biopsy, are still used for the diagnosis of peripheral lung lesions.

The present retrospective study compared the detection rates of different diagnostic methods for PPLC with the view of improving PPLC diagnosis.

## Materials and Methods

### Subjects

Patients with pulmonary peripheral lesions and a suspected diagnosis of lung cancer treated at our hospital between May 2017 and November 2019 were retrospectively analysed. All patients were diagnosed with PPLC using fibreoptic bronchoscopy, CT-PNB, surgical biopsy, or other single or combined examination methods. A comparison of the positive rates of lung cancer obtained using the different methods was made, with respect to a number of factors (including gender, lesion size and location).

This study was conducted in accordance with the Declaration of Helsinki and approved by the ethics committee of our hospital. Patients or family caretakers signed informed consent for the procedures.

### Inclusion and Exclusion Criteria

*Inclusion criteria:* (1) Patients with a chest CT showing peripheral pulmonary nodules, masses, patchy shadows and ground-glass shadows; (2) patients with a highly-suspected diagnosis of lung cancer according to his/her medical history and auxiliary examination; and (3) patients without contraindications associated with fibreoptic bronchoscopy or puncture examination.

*Exclusion criteria:* (1) Patients with highly-suspected metastatic lung cancer, lymph node metastasis, or other signs of metastasis; tuberculosis or other pulmonary infectious diseases; sarcoidosis; or lymphoma; (2) patients with severe cardiopulmonary failure, abnormal coagulation function and other operation contraindications; and (3) patients whose complete clinical data could not be obtained.

### Fiberoptic Bronchoscopy Sampling Methods

*Method of local anaesthesia:* A volume of 5 ml of normal saline was added, and 33.3 mg of tetracaine for injection was sprayed into the nasal cavity and tongue base several times. Then, 5 ml of lidocaine was extracted and inhaled through the nasal cavity several times using a 5-ml syringe connected to a scalp needle tube. Preliminary evaluation of the anaesthetic effect looked for an absence of nausea reaction, swelling sensation, or foreign body sensation in the pharynx. After satisfactory anaesthesia, venous access was established, fibreoptic bronchoscopy was performed, intraoperative oxygen was administered, and the vital signs were monitored.

As the tracheoscope arrived at the subsegment, a TBLB was performed by inserting biopsy forceps into the subsegment according to the CT positioning. If the biopsy forceps encountered resistance, they were pulled back 1–2 cm to open on patient inhalation and close at the end of exhalation. If the patient felt any pain, the biopsy forceps were slowly withdrawn.

Conventional forceps were used to obtain three to five pieces of lung tissue, and the specimens were fixed with a 10% formalin solution and sent to pathology. The brush was then extended into the subsegment, which was brushed three to four times when there was obvious resistance. After the brushing, two samples were made. One piece was subjected to cytological rapid field evaluation (C-Rose) *via* Liu’s staining (8), and the other piece was fixed and sent to the Department of Pathology of our hospital for histopathological examination (9). The different methods for sampling could be combined and used simultaneously as well as independently for any one of three methods.

*Liu’s staining steps:* LiuA dye drops were added to the fixed tissue sections and allowed to stand for 30–45 s; LiuB dye drops were added (LiuA × 2), mixed with air and allowed to stand for 60–90 s. The residual dye was washed off with a slow flow of water, and the stain was observed under a microscope after air-drying.

A quick on-site evaluation was conducted by the pathologist (10–14). If the C-Rose was positive, the operation was completed after the original site was rinsed, and the mirror body, bristles and biopsy forceps were rinsed and sent for histopathological examination. If the C-Rose was negative, the operating position was replaced, and the above steps were repeated in another or several other subsections.

### Sampling Methods for CT-PNB

Patients with negative fibreoptic bronchoscopy results were subjected to a CT-PNB. An appropriate position was taken according to the lesion location shown on the CT. Then, CT scanning positioning was performed to determine the puncture point, angle and route, and the distance between the distal, proximal and pleura of the lesion and the puncture point was calculated to determine the insertion depth.

Routine disinfection, towel-laying and 2% lidocaine 5-ml layer-by-layer infiltration anaesthesia to the pleura were carried out. The puncture needle entered the lung following the established path; when it reached the pre-set lesion location, CT scanning was performed to determine that the tip was at the edge of the lesion. A tissue biopsy was taken by the needle core, which was quickly pulled out after sampling. CT scanning was performed again to determine the presence of intra-pulmonary haemorrhage, pneumothorax, or other complications. The specimen was fixed with a 10% formalin solution for histopathological examination.

### Surgical Biopsy Sampling Methods

Patients without a diagnosis after both fibreoptic bronchoscopy and CT-PNB and with a highly suspected lung cancer diagnosis had to undergo a surgical biopsy or were referred to other hospitals for a further workup. The histological type and TNM staging data were collected after diagnosis.

### Main Observational Index

The main observational index included the detection rates of peripheral lung cancer with different sized lesions by bronchoscopy, brushing, rinsing and CT-PNB.

### Statistical Analysis

The SPSS 21.0 (IBM, Chicago, USA) software was used to conduct the statistical analysis. The continuous variables of normal distribution were expressed as mean ± standard deviation, the continuous variables of non-normal distribution were expressed as median (interquartile range), and the categorical variables were expressed as frequency [percentage (%)].

For multiple comparisons, each value was compared using one-way analysis of variance following a Dunnett test when each datum conformed to normal distribution; meanwhile, non-normally distributed continuous data were compared using non-parametric tests. The counting data were tested using a chi-square test. A *P* value of <0.05 was considered statistically significant.

## Results

### General Characteristics

A total of 359 patients were included in the present study. A total of 186 were examined *via* fibreoptic bronchoscopy; these patients comprised 115 males aged 32–76 years (average age of 59.5 years) and 71 females aged 31–76 years (average age of 59.7 years). The lesion diameter was 0.8–2 cm in 38 patients, 2–5 cm in 86 patients and >5 cm in 62 patients (**Table 1**).

**Table 1:** Comparison of different sampling methods for different lesions of different sizes under fiberoptic bronchoscopy.

Diameter of focus (cm)	n	Brush inspection	Flush	TBLB	Brush inspection +Flush +TBLB	χ^2^	*P*
		detection rate (%)	detection rate (%)	detection rate (%)	detection rate (%)		
0.8 -2	38	5/38 (13%)	3/38 (8%)	6/38 (16%)	13/38 (34%)	10.223	0.016
2-5	86	17/86 (20%)	15/86 (17%)	38/86 (44%)	59/86 (69%)	63.442	<0.001
>5	62	22/62 (35%)	18/62 (29%)	44/62 (71%)	52/62 (84%)	53.664	<0.001
Total	186	44/186 (24%)	36/186 (19%)	88/186 (47%)	124/186 (67%)	113.553	<0.001

There was a statistical difference of P < 0.05. TBLB, transbronchial lung biopsy.

The remaining 173 patients received CT-PNB; these patients comprised 122 males aged 38–81 years (average age of 65.8 years) and 51 females aged 40–78 years (average age of 62.7 years). The PPLC lesion diameter was 0.8–2 cm in 57 patients, 2–5 cm in 65 patients and >5 cm in 51 patients (**Table 2**).

**Table 2:** Comparison of joint sampling method under fiberoptic bronchoscopy and CT-PNB sampling method.

Group	n	Diameter of focus 0.8-2cm	Diameter of focus 2-5cm	Diameter of focus >5cm
		number of positive cases/cases	number of positive cases/cases	number of positive cases/cases
Brush inspection +flush +TBLB	186	13/38 (34%)	59/86 (69%)	52/62 (84%)
CT-PNB	173	47/57 (82%)	61/65 (94%)	49/51 (96%)
χ^2^		22.808	12.457	4.393
*P*		<0.001	<0.001	0.036

There was a statistical difference of P < 0.05. CT, PNB. CT-guided percutaneous lung puncture biopsy; TBLB, transbronchial lung biopsy.

The most common histological type of primary peripheral cancer in both groups was adenocarcinoma (52%), affecting 120 males and 69 females aged 31–81 years (average age of 61.7 years); the second most common cancer was squamous cell carcinoma (27.5%), affecting 69 males and 31 females aged 32–80 years (average age of 61.4 years).

**Figure 1:**
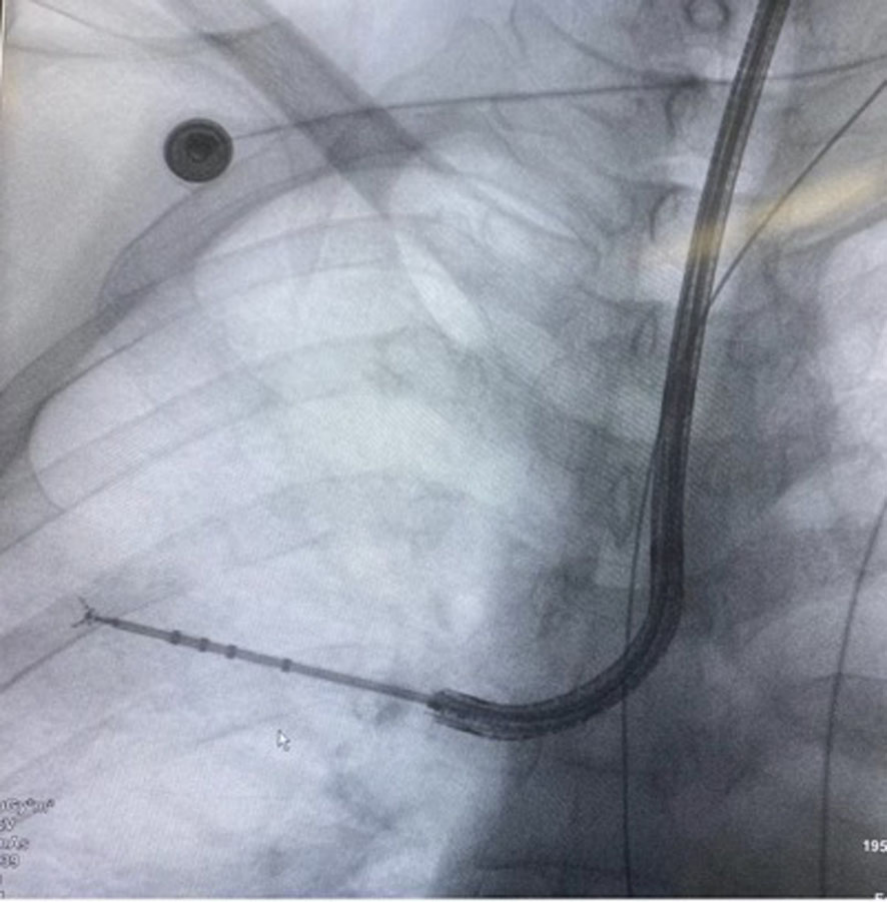
A case of tracheoscopic operation.

**Figure 2:**
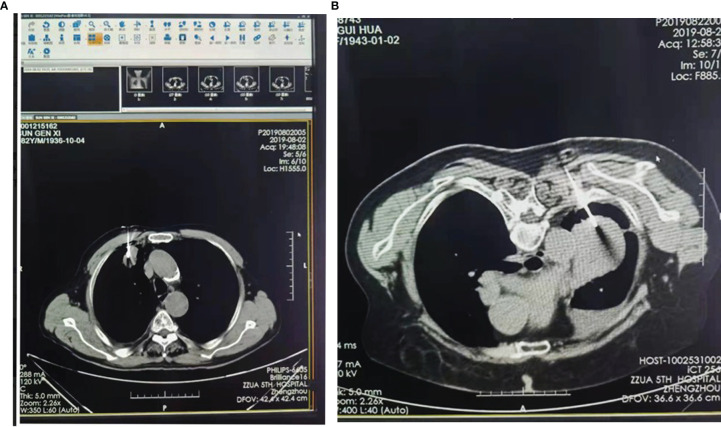
Two case of CT-guided lung puncture. **(A)** Lesion diameter of 2-5 cm; **(B)** Lesion diameter >5cmssssss.

There were no statistically significant differences in clinical features between the fibreoptic bronchoscopy group and the CT-PNB group (*P* > 0.05; **Table 3**). The tracheoscopic operation images are shown in **Figure 1**, and the CT-guided lung puncture images are shown in **Figure 2**.

**Table 3:** Comparison of clinical features between the two groups.

Characteristics		Brush inspection +flush +TBLB	CT-PNB
		N = 186	N = 173
Age (years)			
	≤55	78	67
	>55	108	106
Gender			
	Male	115	122
	Female	71	51
Histological type			
	Squamous cell	53	46
	carcinoma		
	Adenocarcinoma	94	96
	Small cell carcinoma	39	31
TNM Classification			
	I-IIIa	63	70
	IIIb-IV	84	72

CT-PNB, CT-guided percutaneous lung puncture biopsy; TBLB, transbronchial lung biopsy.

### Comparison of Detection Rates

Among the 186 patients who underwent fibreoptic bronchoscopy, 124 produced specimens that were confirmed as peripheral lung cancer, with an overall detection rate of 67%. The positive detection rate of the combined sampling was 34% in lesions with a diameter of 0.8–2 cm, 69% in lesions with a diameter of 2–5 cm and 84% in lesions with a diameter of >5 cm.

The detection rate order of the various sampling methods from highest to lowest was (1) brush detection + irrigation + TBLB, (2) TBLB only, (3) brush inspection and (4) flushing. The differences were statistically significant (*P* < 0.05). The results are shown in **Table 1**.

An analysis of the independent use of the three methods was conducted; the results showed that when the lesion diameter was 0.8–2 cm, there were no significant differences among the detection rates of the brushing, flushing and TBLB. However, when the lesion diameter was 2–5 cm or >5 cm, there were significant differences among the detection rates of the three methods (*P* < 0.05; **Table 4**).

**Table 4:** Comparison of different sampling methods for different lesions of different sizes under fiberoptic bronchoscopy.

Diameter of focus (cm)	n	Brush inspection	Flush	TBLB	χ^2^	*P*
		detection rate (%)	detection rate (%)	detection rate (%)		
0.8 -2	38	5/38 (13%)	3/38 (8%)	6/38 (16%)	1.14	0.565
2-5	86	17/86 (20%)	15/86 (17%)	38/86 (44%)	19.095	<0.001
>5	62	22/62 (35%)	18/62 (29%)	44/62 (71%)	25.529	<0.001
Total	186	44/186 (24%)	36/186 (19%)	88/186 (47%)	40.062	<0.001

There was a statistical difference of P < 0.05. TBLB, taransbronchial lung biopsy.

Hence, the combined detection method had the best detection rate, and TBLB was more reliable than the other two methods.

Among the remaining 62 patients, 30 underwent CT-PNB, 30 underwent surgical thoracoscopic biopsy, and 2 underwent ENB + R-EBUS + GS; all were confirmed with lung cancer.

Among the 173 patients who underwent CT-PNB examination, 157 had a histopathological confirmation of lung cancer. The positive detection rate was 82%, 94% and 96% when the lesion diameter was 0.8–2 cm, 2–5 cm and >5 cm, respectively (i.e. the positive detection rate in peripheral lesions with a diameter of <5 cm was significantly higher in CT-PNB than in tracheal microscopy; the difference was statistically significant (*P* < 0.05; **Table 2**). The other three unconfirmed cases were confirmed as lung cancer *via* thoracoscopic biopsy.

## Discussion

The early diagnosis of PPLC is a problem often encountered by pulmonary physicians (15). Fibreoptic bronchoscopy and microfiber bronchoscopy can generally observe the level of grade 3–4 and 4–6 bronchial tubes, respectively. Hence, these methods can be used to give a clear diagnosis in 70% of patients with lung cancer and are thus widely used in early lung cancer screening and diagnosis (16). However, the majority of peripheral lesion cases involve blind examinations, which greatly reduces the positive rate of the biopsy, especially with small lesions.

In the present study, the positive detection rates of CT-PNB were 82%, 94% and 96% in PPLC lesion diameters of 0.8–2 cm, 2–5 cm and >5 cm, respectively. These rates were higher than in tracheal microscopy; the difference was statistically significant (*P* < 0.05).

With peripheral lesion diameters of >5 cm, the positive rate of fibreoptic bronchoscopy was 84%. Using a combination method (e.g. brush inspection + flushing + TBLB) is superior to using a single one. Therefore, when a peripheral lesion is small, CT-PNB can be used, and when the lesion is larger, either a bronchoscope examination or CT-PNB can be chosen depending on the patient’s condition.

CT can help predict the PPLC type, locate the lesion site, plan the puncture path and depth and perform fine needle punctures; these are less invasive, have a high positive detection rate and are easy and fast to operate (17–21).

When a lesion is close to the lung and hilum, especially an endobronchial lesion, fibreoptic bronchoscopy biopsy and brush examination are preferred; however, when a lesion is close to the outer lung and chest wall, performing fibreoptic bronchoscopy is difficult (22).

However, CT-PNB can improve the diagnostic rate of peripheral lung diseases, and the combination of the two has a higher positive detection rate. Although CT-PNB requires a higher pain tolerance than fibre bronchoscopy, the largest number of patients to consider is those undergoing radiation (23).

At present, many patients can take advantage of the navigation technology of pulmonary peripheral lesions, such as R-EBUS and ENB; this can make up for the inadequacy of fibre bronchoscopy, and radiation does not have to be considered for the patients (7). However, the new technology is not yet very popular; some of it is expensive, restricting its promotion to a certain extent.

The C-Rose technique (24) was also used in the present study. This technique involves cytological staining, interpretation, rapid evaluation and preliminary diagnosis of specimens obtained by puncturing and other methods at a sampling site. In recent years, more and more attention has been paid to the C-Rose technique, which can be used in combination with various respiratory interventions, such as bronchoscopy and percutaneous lung puncture (25).

The roles of C-Rose are mainly the following: (1) to improve the quality of minimally invasive specimens, reduce the number of punctures, improve the positive detection rate of surgical operations and reduce complications; (2) to allow for a rapid diagnosis so that treatment can be started as early as possible; and (3) to improve the utilisation rate of specimens (25).

In addition, the only equipment required are microscopes and dyeing reagents. Therefore, promoting the technique’s use in respiratory endoscopic intervention centres is recommended.

However, all current endoscopic imaging techniques, including endoscopic ultrasound, autofluorescence and optical coherence tomography and laser confocal microscope endoscopy, have a number of limitations (26–28).

Although ultrasonography has a strong penetrability, its resolution is low, and its application in gas-rich lungs is greatly restricted (29). It is also difficult to diagnose diseases with ground-glass shadows or without a bronchial pathway through imaging. The resolution of confocal microscopy (26, 30) can reach 1 µm, and the nucleus can be clearly resolved; however, the field of vision is very small, so it may take a long time to find the lesion site.

Application of non-invasive methods for disease diagnosis is the ultimate goal; however, it is difficult to do so at present, and the use of a biopsy is still of decisive significance (31).

For peripheral lung lesions, both benign and malignant lesions should be distinguished in addition to the tumour type, stage and genetic background, all of which require histopathological specimens. It is important to study the relationship between imaging and lesion properties, but biopsy is still irreplaceable at this stage (31).

The present study has several limitations. Firstly, it is only a retrospective study; secondly, it is a single-centre study, limiting the exploration of patterns across different setups; and thirdly, the sample size is relatively small, allowing for potential large deviations in some of the results.

## Conclusion

In the diagnosis of peripheral lung cancer, CT-PNB had the highest detection rate in peripheral lesions with a diameter of <5 cm. It was also found that the combination method (brush inspection + flushing + TBLB) was superior, regardless of the lesion size. Therefore, when a peripheral lesion is small, CT-PNB can be used; meanwhile, according to the patient’s condition, either a bronchoscope examination or CT-PNB can be used for larger lesions.

## Data Availability Statement

The original contributions presented in the study are included in the article/supplementary material. Further inquiries can be directed to the corresponding author.

## Ethics Statement

This study was conducted with approval from the ethics committee of The Fifth Affiliated Hospital of Zhengzhou University. The patients/participants provided their written informed consent to participate in this study.

## Author Contributions

LS has made substantial contributions to conception and design. DG, acquisition of data, analysis and interpretation of data. LS, QF, and WZ have been involved in drafting the manuscript and revising it critically for important intellectual content. SH and HL have given final approval of the version to be published.

## Conflict of Interest

The authors declare that the research was conducted in the absence of any commercial or financial relationships that could be construed as a potential conflict of interest.

## Publisher’s Note

All claims expressed in this article are solely those of the authors and do not necessarily represent those of their affiliated organizations, or those of the publisher, the editors and the reviewers. Any product that may be evaluated in this article, or claim that may be made by its manufacturer, is not guaranteed or endorsed by the publisher.
